# Fabrication of hierarchical core/shell MgCo_2_O_4_@MnO_2_ nanowall arrays on Ni-foam as high–rate electrodes for asymmetric supercapacitors

**DOI:** 10.1038/s41598-019-48931-6

**Published:** 2019-08-29

**Authors:** Jiasheng Xu, Lin Wang

**Affiliations:** 1 College of Chemistry, Chemical Engineering and Environmental Engineering, Liaoning Shihua University, Fushun, 113001 P.R. China; 2 Liaoning Province Key Laboratory for Synthesis and Application of Functional Compounds, College of Chemistry and Chemical Engineering, Bohai University, Jinzhou, 121013 P.R. China

**Keywords:** Nanoparticles, Supercapacitors

## Abstract

Design and fabrication of a hierarchical core/shell MgCo_2_O_4_@MnO_2_ nanowall arrays on Ni-foam by a facile two-step hydrothermal method. The electrochemical measurements prove these composites with MnO_2_ definitely offer better supercapacitive performance of the MgCo_2_O_4_ electrode material. The nanowall structure provides more active sites and charge transfer during the Faradic reaction. The MgCo_2_O_4_@MnO_2_ nanowall shows an excellent electrochemical performance (852.5 F g^−1^ at 1 A g^−1^). The asymmetric supercapacitor is composed of the MgCo_2_O_4_@MnO_2_ nanowall and the activated carbon (AC). The energy densities of the asymmetric supercapacitor device can keep up 67.2 Wh·kg^−1^ at 5760.0 W·kg^−1^. The MgCo_2_O_4_@MnO_2_ nanowall shows excellent supercapacitive performance and has a great potential for more research and application in the asymmetric supercapacitor devices field.

## Introduction

With the increase of environmental pollution and the growing energy consumption, the research on new energy and energy storage device is very urgent^1–3^. Some new energy storage devices enter the field of view of researchers, such as fuel cells, Li-ion batteries and electrochemical capacitors^4–7^. The novel energy storage device is typically environmentally friendly, reusable and high conversion efficiency^8–10^. Right now, the interest in electrochemical capacitors (which also called supercapacitors) is growing worldwide. Supercapacitors are used in electric vehicles, computer memory system audio equipment and intermittent power supply systems^11–13^. The high energy storage device is mainly composed of electrodes and electrolyte^14–17^. The researches of the materials are significant for the development of supercapacitors^18–23^.

The special crystal structure of the spinel-type oxides gathers enthusiasm for research in recent years. The ordered microstructure of the spinel-type oxides provides the stable electrochemical performance^24,25^. The AB_2_O_4_ is a typical ternary oxide, has been widely researched as a high-rate anode material for supercapacitors^26,27^. The transition metal oxides were deemed to be excellent as the electrode materials for reaction pseudocapacitance. The transition metal element provides more potential charge transfer transitions^28^. The ligand-to-metal charge-transfer transition can easily occur in the high oxidation transition metal oxides^29–31^. These materials usually have excellent electrochemical performance which has the AB_2_O_4_ spinel with two transition metal elements and one of the elements is cobalt^32–39^. The cobalt-based oxides with AB_2_O_4_ spinel structure are widely used in the field of supercapacitors, such as NiCo_2_O_4_^40^, ZnCo_2_O_4_^20^, CuCo_2_O_4_^41^ are better electrode materials for supercapacitors. The MgCo_2_O_4_ (a typical AB_2_O_4_ spinel-type structure) has a theoretic specific capacitance (3122 F g^−1^)^29,42,43^. This stable structure of MgCo_2_O_4_ will lead to a new research field. MnO_2_ (high theoretical capacity ~1370 F g^−1^) as one of the supercapacitor electrode materials has been extensively investigated. MnO_2_-based nanocomposites can be used in aqueous electrolytes, which can meet the requirements of the test conditions^44–46^. A feasible attractive design is to grow MnO_2_ nanostructures on MgCo_2_O_4_ nanosheets by two steps of hydrothermal to get the MgCo_2_O_4_@MnO_2_ core/shell structure.

Herein, the MgCo_2_O_4_ nanowall arrays (MCNA) and the hierarchical core/shell MgCo_2_O_4_@MnO_2_ nanowall arrays (MCMNA) have fabricated in the series of the processes. The as-prepared MgCo_2_O_4_ nanowall arrays are using a hydrothermal reaction. The nanowall (on the MgCo_2_O_4_@MnO_2_) was prepared using the second mild step of the hydrothermal reaction. The pore volume of the MgCo_2_O_4_@MnO_2_ is 0.69 cm^3^·g^−1^ and the surface area is 140.04 m^2^·g^−1^. The typical nanowall thickness is about 100 nm, the microstructure of the nanosheets are regular and dense. These nanostructures of the MCMNA-2 provide the rich reactive compared with MCNA, MCMNA-1 and MCMNA-3. The MCMNA-2 sample shows a prominent property at 1 A g^−1^ that the specific capacity is 852.5 F g^−1^ and it shows the excellent cycle stability after 2000 cycles. Table S1 shows the compared MCMNA-2 with other reported. The energy densities of the MCMNA-2//AC asymmetric supercapacitor device can keep up 67.2 Wh·kg^−1^ at 5760.0 W·kg^−1^.

## Results and Discussion

### Schematic of the fabrication procedure for the core/shell MgCo_2_O_4_@MnO_2_ nanowall arrays on Ni-foam

**Figure 1:**
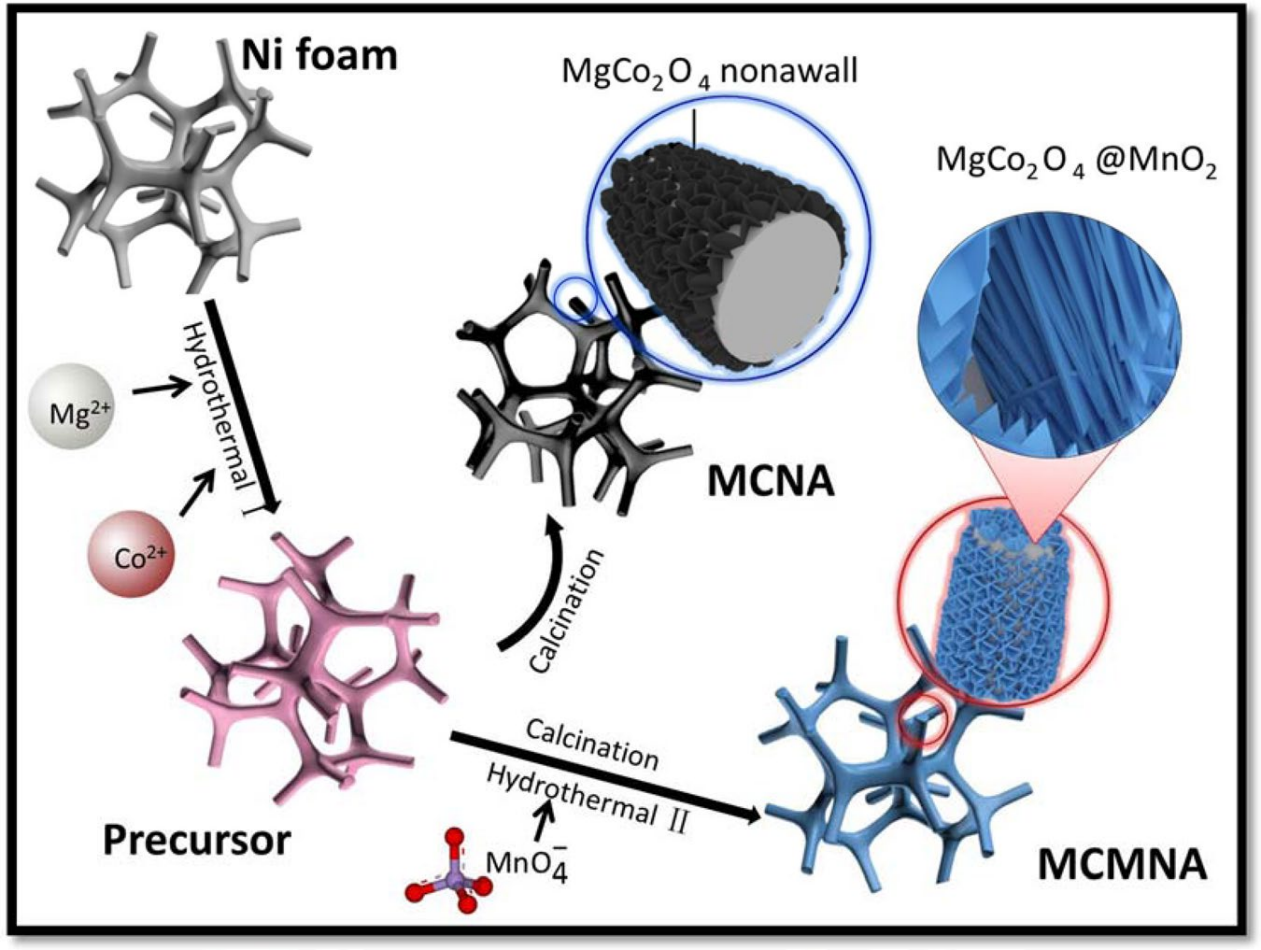
Schematic illustrating the fabrication procedure of the MgCo_2_O_4_@MnO_2_ core-shell nanowall arrays on Ni foam.

The core/shell MgCo_2_O_4_@MnO_2_ nanowall arrays (MCMNA) on Ni-foam were prepared via two steps of hydrothermal reaction. The fabrication procedure of the MCMNA electrode is schematic illustrating in Fig. 1. After the first hydrothermal reaction, Mg ions react with Co ions to form a pink MgCo_2_O_4_ nanowall arrays (MCNA) layer on Ni-foam. The MnO_2_ sheets are grown on the ordered MCNA in the second hydrothermal reaction.

### Characterization of the as-prepared MgCo_2_O_4_ nanowall arrays MgCo_2_O_4_@MnO_2_ core/shell nanowall arrays

**Figure 2:**
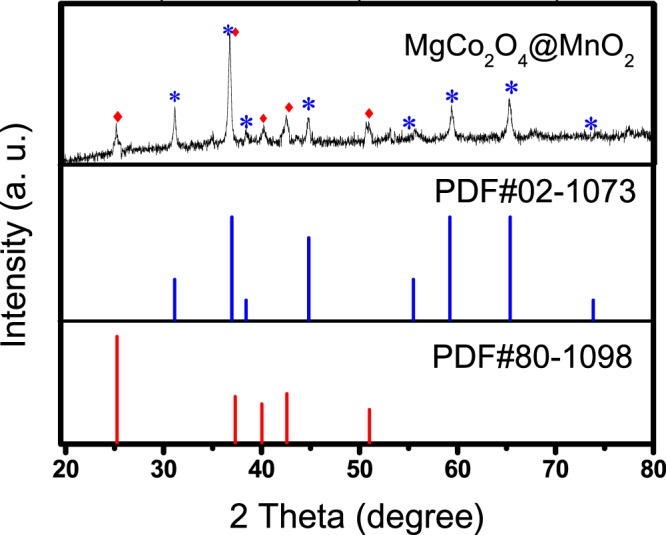
Typical XRD patterns of the hierarchical MgCo_2_O_4_@MnO_2_ core-shell nanowall arrays, which corresponds well to the standard diffraction pattern of MgCo_2_O_4_ (PDF card No. 02-1073) and MnO_2_ (PDF card No. 80–1098). The peaks generated by MgCo_2_O_4_ are marked with stars; the peaks generated by MnO_2_ are marked with diamond signs.

In order to analyze the composition and crystal phase details, the hierarchical MgCo_2_O_4_@MnO_2_ core/shell nanowall arrays was characterized by XRD. The typical XRD patterns (from 20° to 80°) of the hierarchical core/shell MgCo_2_O_4_@MnO_2_ nanowall arrays is shown in Fig. 2 shows. The well-defined diffraction peaks are evident in these XRD patterns. These diffraction peaks (marked with a star sign) correspond well to the characteristic peaks of spinel MgCo_2_O_4_ phase (PDF card No. 02-1073), which are (220), (311), (222), (400), (422), (511), (440), (620) and (444), respectively. Other reflection peaks (marked with a diamond sign) which are correspond to the PDF card (No. 80–1098) characteristic peaks of MnO_2_. The diffraction peaks (low and wide) of the hierarchical core/shell MgCo_2_O_4_@MnO_2_ nanowall arrays show a low crystallinity, indicating that the crystallite has a small size.

**Figure 3:**
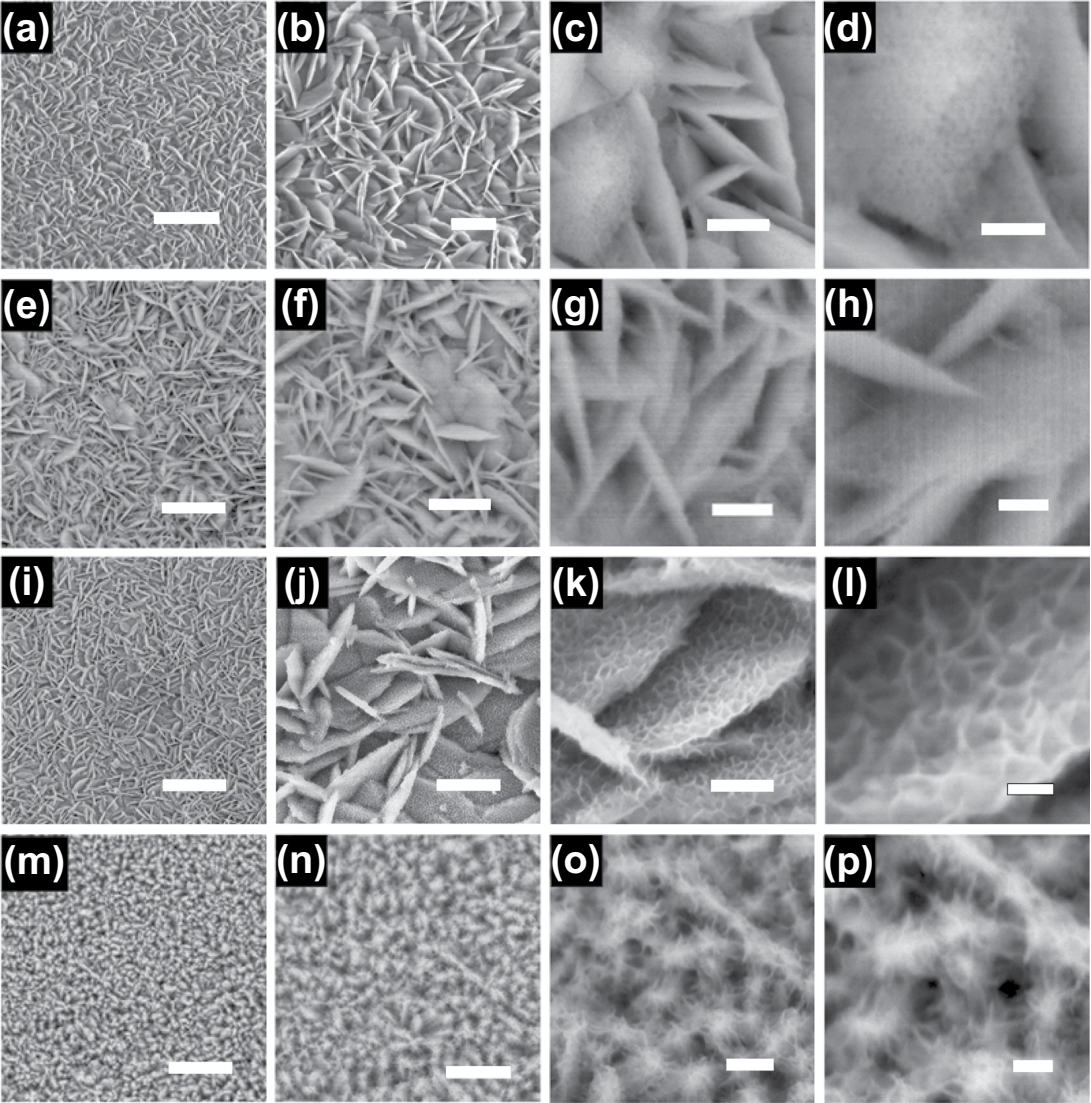
SEM images of the MgCo_2_O_4_ nanowall arrays on Ni-foam and the SEM images of the hierarchical MgCo_2_O_4_@MnO_2_ core-shell nanowall arrays on Ni-foam at different reaction time in the second step hydrothermal process: (**a**–**d**) the different magnification SEM images of the MgCo_2_O_4_ nanowall arrays on Ni-foam (MCNA), scale bars, 4 μm, 1 μm, 200 nm and 100 nm, respectively; (**e**–**h**) SEM images of the MgCo_2_O_4_@MnO_2_ core-shell nanowall arrays (MCMNA-1) on Ni foam, scale bars, 2μm, 1 μm, 300 nm and 200 nm, respectively; (**i**–**l**) SEM images of the MgCo_2_O_4_@MnO_2_ core-shell nanowall arrays on (MCMNA-2) Ni foam, scale bars, 2 μm, 1 μm, 500 nm and 200 nm, respectively; (**m**–**p**) SEM images of the MgCo_2_O_4_@MnO_2_ core-shell nanowall arrays (MCMNA-3) on Ni foam, scale bars, 2 μm, 1 μm, 500 nm and 200 nm, respectively.

Figure 3a–d show the SEM images of MgCo_2_O_4_ nanowall arrays (MCNA). The SEM images of MgCo_2_O_4_@MnO_2_ nanowall arrays (MCMNA) (which have different second hydrothermal steps) are shown in Fig. 3e–p. In Fig. 3a,b, the low magnification SEM images show the uniform nanostructures and morphologies of MCNA. A panoramic morphology in Fig. S1 shows the Ni-foam completely covered by MCNA. The high magnification SEM images (in Fig. 3c,d) show the uniform thickness sheets of the MgCo_2_O_4_ nanowall arrays is about 20 nm. It is found that the MCNA are vertically grown on the surface of the Ni-foam skeleton and interconnected with each other. The new micro/nanostructure of the MCNA can provide a high-rate reaction surface for electrolytes and the electrodes. The SEM images in Fig. 3e–h show the core/shell MgCo_2_O_4_@MnO_2_ nanowall arrays on Ni-foam which with the second hydrothermal with 120 °C for 2 h (MCMNA-1). The SEM images on low magnification (Fig. 3e,f) of the MCMNA-1 are similar to the SEM images of the MgCo_2_O_4_ nanowall arrays. The high magnification SEM images of the MCMNA-1 are shown in Fig. 3g,h, where the Fig. 3h is a further amplification of the Fig. 3g. It can be seen that there are a bit of MnO_2_ on the nanowall in the Fig. 3g,h. The SEM images in Fig. 3i–l show the core/shell MgCo_2_O_4_@MnO_2_ nanowall arrays on Ni-foam which with the second hydrothermal steps at 120 °C for 4 h (MCMNA-2). There are many regular nanowall grown on Ni-foam which are shown in low magnification SEM images (Fig. 3I,j). The thickness of the MCMNA-2 on Ni-foam is about 70 nm (shown in Fig. 3k,l). The thickness of the nanowall was increased due to the long second hydrothermal treatment time. The MCMNA-2 can provide a larger charge-discharge reaction surface area than MCMNA-1. The core/shell structure of the MCMNA-2 offered electrochemical reaction the ionic transmission in electrolytes and electrode. The SEM images of the core/shell MgCo_2_O_4_@MnO_2_ nanowall arrays (MCMNA-3) are shown in Fig. 3m–p. With the increase of the second hydrothermal treatment time to 6 h (at 120 °C), the microscopic morphology of the MnO_2_ shell structure continued to change (compared with Fig. 3d,h,l). Figure 3m,n are the low magnification SEM images. Figure 3o,p are the high magnification SEM images of the MCMNA-3 on Ni-foam. In Fig. 3o,p, it is observed that the typical nanowall thickness is about 100 nm. The MnO_2_ shell of the MCMNA-3 is obviously thicker than these of MCMNA-1 and MCMNA-2. The long second hydrothermal treatment time can effectively promote the MnO_2_ grown on the MgCo_2_O_4_ nanowall. The SEM images on high magnification (Fig. 3p) exhibit that the MnO_2_ nanowall grows more with the reaction time increasing. The nanosheets of the MCMNA-3 are almost completely covering the MgCo_2_O_4_ nanowall core.

**Figure 4:**
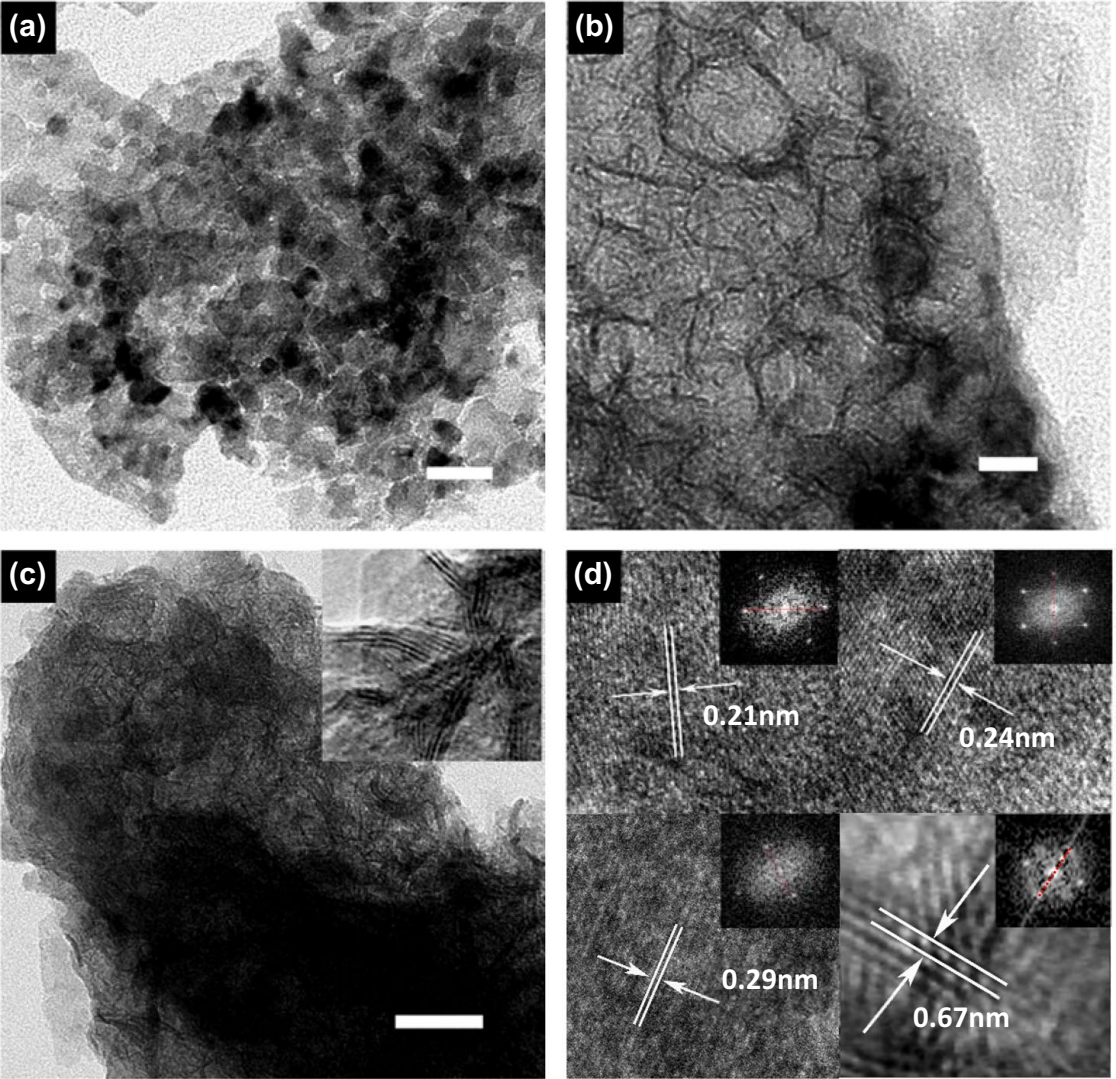
(**a**) TEM images of the MCNA, scale bars = 30 nm; (**b**) TEM images of the MCMNA-2, scale bars = 20 nm (**c**) TEM images of the MCMNA-2, scale bars = 50 nm, inset is HRTEM images of the MCMNA-2. (**d**) HRTEM images of the MCMNA-2 (the inset is a Fourier image of calculated using Fourier transform.) show the present MgCo_2_O_4_ and MnO_2_ phase.

The morphology and structure of MCNA and MCMNA-2 were further characterized using transmission electron microscopy (TEM) analyses. The TEM and the HRTEM images of the MCNA and MCMNA-2 are shown in Fig. 4. The low magnification TEM image of MCNA exhibits distributed uniformly (Fig. 4a). It is clear that the MCNA composed form of the amounts of nanoparticles which increased the facilitate of the electrolyte penetration and surface area. In Fig. 4b, the TEM images of MCMNA-2 are consisted of stacking MnO_2_ nanosheets distributed the MgCo_2_O_4_ nanowall substrates, which appears the uniform structure and the size agrees well with the SEM images. In Fig. 4c, the typically covered MnO_2_ nanowalls on the surface of the MCNA substrate issue in the core/shell nanostructure. The core/shell nanostructure can provide a better environment in reactions for the fast transportation of ions and electrons.

The inset in Fig. 4c is the high magnification TEM images of the MCMNA-2, which can further exhibit the details and the structure of the covered MnO_2_ nanosheets. Figure 4d shows the HRTEM images of the MCMNA-2, the corresponding inset is the Fourier images which are calculated using Fourier transform. The interplanar spacing was computed at 0.21 nm, 0.24 nm and 0.29 nm (Fig. 4d), corresponding well to the distances of (021), (212) and (111) plane of the MgCo_2_O_4_, respectively. The lattice spacing of 0.67 nm (in Fig. 4d) correspond well with the (001) plane of the typical MnO_2_. The results of the calculation from the HRTEM analysis accord with XRD analysis (as the Fig. 2).

**Figure 5:**
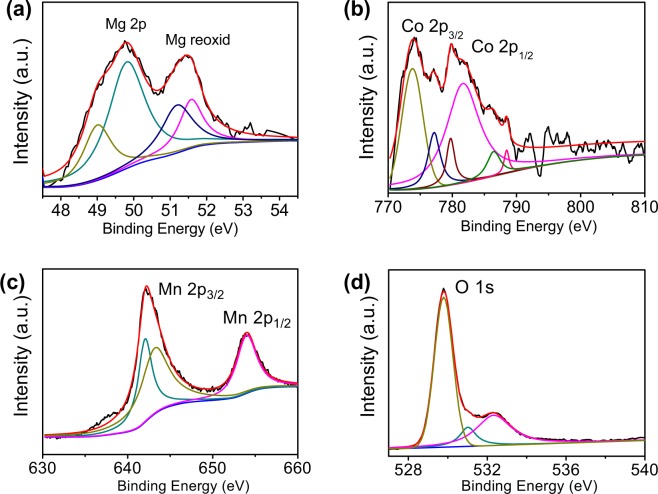
XPS spectra of the MgCo_2_O_4_@MnO_2_ core-shell nanowall arrays. (**a**) Mg 2p spectrum; (**b**) Co 2p spectrum; (**c**) Mn 2p spectrum; (**d**) O 1 s spectrum.

X-ray spectroscopy photoelectron (XPS) is the typical quantitative spectroscopic science method for identifying the valence states of the elements in the core/shell MgCo_2_O_4_@MnO_2_ nanowall. Figure 5 shows the XPS spectra of the MgCo_2_O_4_@MnO_2_. The spectra show the presence of Mg, Co, O and Mn. Fig. S4 shows the entire XPS spectra of the MgCo_2_O_4_@MnO_2_. In the spectrum of the Mg 2p, the obvious binding-energy of the photoelectron peaks at 49.0, 49.8, 51.2 and 51.6 eV are revealed in Fig. 5a. The obvious binding-energy of the photoelectron peaks at 49.0 and 49.8 eV with the separation-energy of 0.8 eV belong to Mg 2p energy level of MCMNA. The obvious binding-energy of the photoelectron peaks at 51.2 and 51.6 eV accord with the characteristic of Mg^2+^ oxidation state from the MCMNA. The Co 2p spectrum shows two peaks at 774.0, 777.2, 779.8 and 781.8 eV with a binding-energy separation of 7.8 eV, which accord with the Co 2p_3/2_ and Co 2p_1/2_ (Fig. 5b) energy level in MgCo_2_O_4_@MnO_2_, respectively. The spectrum appears has a higher binding-energies in the wide feature centered at 103.67 eV. The spectrum of the O 1 s exhibits a peak at binding-energy of 529.8 eV, the characteristic bands corresponding to an oxygen atom. Another peak (binding-energy of 531.7 eV) corresponds to the characteristic bands of an oxygen atom (with a hydroxyl group).

Fig. S5 shows the Brunauer-Emmett-Teller (BET) result of the hierarchical core/shell MgCo_2_O_4_@MnO_2_ nanowall. The isothermal plots of N_2_ adsorption/desorption for the hierarchical core/shell MgCo_2_O_4_@MnO_2_ nanowall arrays with a clear hysteresis loop show the pours nanostructure. In Fig. S5, the desorption curve (marked with squares) and the adsorption curve (marked with circles) compose the IV typical isotherm. It is a typical type adsorption/desorption isotherms in the six classifications recommended by IUPAC, which can be attributed to the middle part of the H3 type adsorption hysteresis loop. Insert shows the pore size distribution of the hierarchical core/shell MgCo_2_O_4_@MnO_2_ nanowall arrays. The BET surface area of the hierarchical core/shell MgCo_2_O_4_@MnO_2_ nanowall arrays is about 140.04 m^2^·g^−1^ and the pore volume is 0.69 cm^3^·g^−1^. The average pore size computed using by BJH model of 31 nm, and most pore sizes range from 10 to 40 nm, and. This hierarchical core/shell MgCo_2_O_4_@MnO_2_ nanowall arrays has a large pore volume and a high reaction area which is beneficial to the high-rate charge/discharge reactions.

### Electrochemical measures of the prepared MgCo_2_O_4_ nanowall arrays and core/shell MgCo_2_O_4_@MnO_2_ nanowall arrays

**Figure 6:**
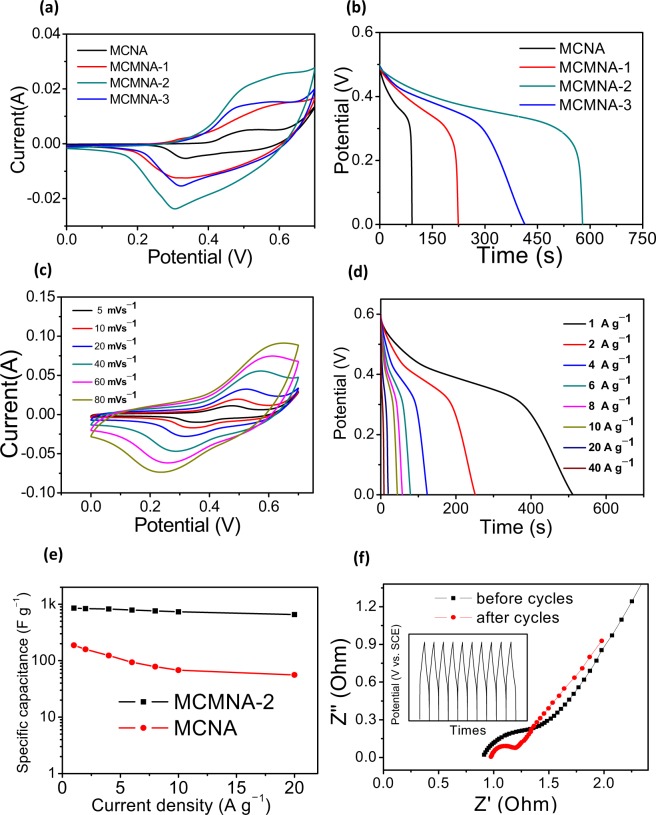
Electrochemical characterizations of MgCo_2_O_4_@MnO_2_ core-shell nanowall arrays. (**a**) Cyclic voltammetry (CV) curves of MCNA, MCMNA-1, MCMNA-2 and MCMNA-3 at 20 mV s^−1^. (**b**) Galvanostatic charge-discharge (GCD) curves of MCNA, MCMNA-1, MCMNA-2 and MCMNA-3 with 1 A g^−1^ at 0.5 V. (**c**) Cyclic voltammetry (CV) curves of the MCMNA-2 at the different scan rates from 5 to 80 mV s^−1^. (**d**) Galvanostatic charge-discharge (GCD) curves of MCMNA-2 at different current densities from 1 to 40 mA g^−1^. (**e**) The specific capacity of the MCMNA-2 and MCNA electrodes as a function of current density. (**f**) Nyquist plots of the MgCo_2_O_4_ nanoneedle arrays/Ni-foam electrode before and after 2000 cycles at the open circuit potential. Inset is the typical 10 cycles with 10 A g^−1^ at 0.5 V.

The electrochemical performances of the MCNA, MCMNA-1, MCMNA-2 and MCMNA-3 were tested as the work electrodes in KOH (2 M) aqueous. The Fig. 6 shows the electrochemical performance of MCMNA in this three-electrode system. Especially, the cyclic voltammetry (CV) curves of MCNA, MCMNA-1, MCMNA-2 and MCMNA-3 (as the working electrodes, at a scan rate of 20 mV·s^−1^) are shown in Fig. 6a.

As shown in Fig. S6, the cyclic voltammetry curve of Ni-foam does not have a significant redox peak, and the MCMNA-2 material prepared has a very obvious redox peak. The contribution of Ni-foam to the electrode capacitance can be neglected. The gravimetric capacitance was determined based on the enclosed area of the CV loop. Typical CV curves in Fig. 6a show that each enclosed areas of the MCMNA (MCMNA-1, MCMNA-2, MCMNA-3) electrode is larger than MCNA. Figure 6b shows the discharge curves of the MCNA, MCMNA-1, MCMNA-2, and MCMNA-3, with 1 A g^−1^ at 0.5 V. The discharge time of the electrodes determined the discharge capacity at the same current densities. The discharge curves of the MCMNA-1, MCMNA-2, and MCMNA-3 show longer discharge time than that of the MCNA.

These results obviously indicate the MnO_2_ nanowall (as the shell) provides the added capacitance. In the Fig. 6a, we can see the enclosed area of the MCMNA-2 is the largest. The Fig. 6b shows that the discharge time of the MCMNA-2 is the longest. These results in Fig. 6a,b show that the reaction time of the MnO_2_ composite process is an important factor on the specific capacitance, MCMNA-2 has a high electrochemical property in these four electrodes.

In Fig. 6c, MCMNA-2 tested on the different scan rates from 5 to 80 mV·s^−1^ under window potential of 0–0.7 V. Every typical CV curves in Fig. 6c show the clear peaks anodic and cathodic current for a reversible reaction, respectively. As the scan rate increases, the cathode peak shifts to a positive potential and the anode peak shifts toward a negative potential peak. The pair of redox peaks in Fig. 6c demonstrates that the electrochemical performance (MCMNA-2) is due to the pseudocapacitive behavior.

Figure 6d shows the MCMNA-2 discharge curves of increasing current densities measured in window potential 0–0.6 V of 1, 2, 4, 6, 8, 10, 20 and 40 A g^−1^, respectively. In Fig. 6d, the discharge curves show a platform region which indicated the characteristic of the Faradic reaction. As shown in these curves, the discharge time and the specific capacities decrease with the increasing current densities. The specific capacitances of MCMNA-2 at increasing current densities which are computed by the curves data of the charge-discharge (Fig. 6d), there is the formulae^47,48^:

1 $${C}_{s}=\frac{{I}\times {\Delta }{\rm{t}}}{{m}\times {\Delta }{\rm{V}}}$$

where *C*_*s*_ (F g^−1^) is the specific capacitance; *Δt* (s) is the discharge time; *I* (A) is the current of discharge reaction; *ΔV* (V) is voltage; *m* (g) is the electrode mass.

In Fig. 6d, the GCD is measured in 0–0.5 V with different current densities of 1, 2, 4, 6, 8, 10, 20 and 40 A g^−1^. The MCMNA-2 sample exhibits excellent performances with specific capacity of 852.5 F g^−1^, 837.7 F g^−1^, 824.7 F g^−1^, 788.0 F g^−1^, 762.7 F g^−1^, 735.0 F g^−1^, 656.7 F g^−1^ and 573.3 F g^−1^, respectively.

The specific capacitance of the MCMNA-2 and MCNA at different current densities is displayed in Fig. 6e. The MCMNA-2 as the electrode exhibits better electrochemical performance and higher specific capacitance than the MCNA in Fig. 6e. The calculated results in Fig. 6e demonstrate that the shell MnO_2_ nanosheets provide richer redox reactions in the discharge process.

The electrochemical performance of the MCMNA-2 has been further confirmed by Nyquist plots of the electrochemical impedance spectrum (EIS). In Fig. 6f, the curves show the Nyquist plots of the MCMNA-2 before and after 2000 cycles. The 10 continuous typical GCD cycles curves are shown in Fig. 6f (inset). The MCMNA-2 electrode was tested (open-circuit voltage is 0.005 V) in the frequency region 10^−2^ to 10^5^ Hz. In the electrochemical test system, the Faradic impedance of the KOH-MCMNA interface is composed of the charge transfer resistance and the joint electrical resistance. The EIS curve consists of a semicircle and a straight line in high and low frequency regions, respectively. As shown in Fig. 6f, after 2000 cycles there is minute variation in the Nyquist plots. In the high frequency area, the curve of the after 2000 cycles has almost same Warburg resistances (*W*_0_) with before. The solution resistance (*R*_*s*_) before 2000 cycles is 1.01 Ω and after 2000 cycles is 1.04 Ω. The curves of high frequency shows the excellent electrochemical stability of MCMNA-2. The low frequency show the capacitance resistance was increased with the 2000 cycling. On the whole, the EIS test results further confirm MCMNA-2 electrode has a stable cycle stability.

**Figure 7:**
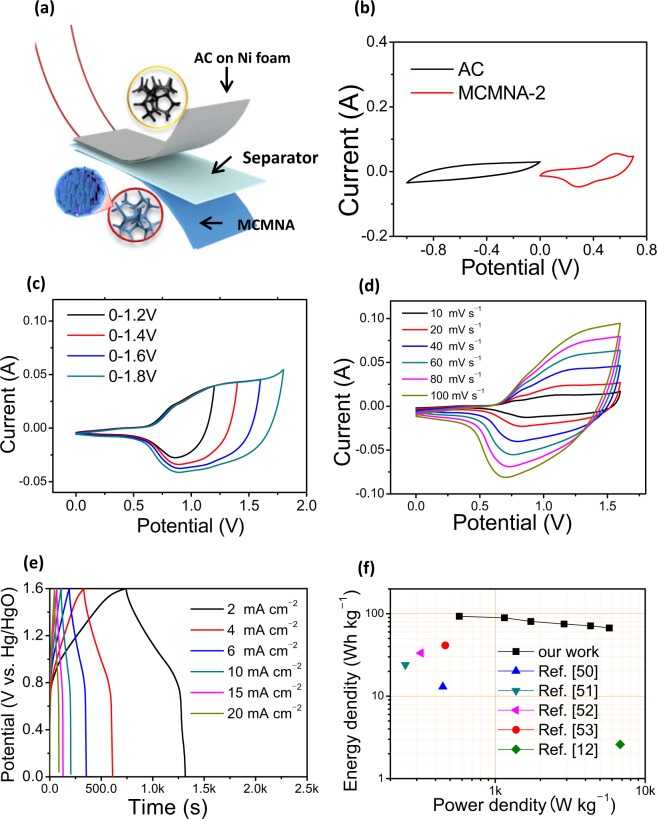
Electrochemical behavior of an asymmetric supercapacitor with the MgCo_2_O_4_@MnO_2_ as the positive electrode, the activated electrode (MCMNA-2//AC device). (**a**) Structural illustration of the MCMNA-2//AC device; (**b**) CV curves of the negative electrode (AC) and the positive electrode (MCMNA-2) at a scan rate 40 mV s^−1^; (**c**) CV curves of the MCMNA-2//AC ASC device at different scan voltage windows at a same scan rate of 40 mV s^−1^; (**d**) CV curves of the MCMNA-2//AC ASC device at different scan rate range from 10 to 100 mV s^−1^; (**e**) GCD curves of the MCMNA-2//AC device at different current densities range from 2 to 20 A g^−1^ under the voltage windows of 0–1.6 V. (**f**) Ragone plots of the MCMNA-2//AC ASC device.

In the cause of the future electrochemical property test of MCMNA-2, the asymmetric supercapacitor (ASC) devices are assembled. The structure illustration of MCMNA-2//AC is shown in Fig. 7a which included three parts. The ASC devices are composed of the positive materials, negative materials, the separator and the electrolyte (PVA/KOH gel). The positive electrode is the MCMNA-2, the negative electrode is the activated carbon (AC) coated on Ni-foam, the separator is a piece of cellulose paper.

Figure 7b shows the CV curves of AC (negative material) and MCMNA-2 (positive material), respectively. The window potential of the negative electrode is form −0.9 V to 0 V and the window potential of the positive electrode is 0 to 0.7 V. It can help determine that the MCMNA-2//AC ASC devices are applicable for the window potential from 0–1.7 V.

A series of tests were performed on the MCMNA-2//AC ASC device to choose the potential window at 40 mV s^−1^ scan rate. Figure 7c shows the increasing potential window (from 0–1.2 V to 0–1.8 V) of the CV curves. The clear redox peaks show that the reaction accompanied by electron charge-transfer in the ASC device. In Fig. 7c, a complete of the redox peaks appear at the test voltage at 0–1.6 V and 0–1.8 V, and the suitable potential window is 0–1.6 V.

Figure 7d shows the CV curves of the MCMNA-2//AC ASC devices at the different scan rates from 10 to 100 mV s^−1^ (0–1.6 V). The oxidation and reduction peaks in Fig. 7d show the ASC device with Faradaic charge/transfer on the interface of electrolyte and the surface of an electrode. The peaks of the cathodic and anodic current appearing in the each curves shows the stability of the ASC device in the electrochemical test.

Figure 7e shows the GCD curves of MCMNA-2//AC ASC in increasing current densities at 2, 4, 6, 8, 10, 15 and 20 A g^−1^ under window potential of 0–1.6 V. The charge and discharge curves shows a platform region which indicated the characteristic of the Faradaic pseudocapacitance. As shown in these curves, the discharge time and the specific capacities decrease as the current density increase.

The energy and the power densities was vary with current, as shown in Fig. 7f. The energy densities and the power densities have been calculated by three formulas^48,49^:

2 $${\rm{\Delta }}Q=\frac{{I}\times {\Delta }{\rm{t}}}{m}$$

3 $${E}_{s}=\frac{1}{2}Q\times {\Delta }{\rm{V}}=\frac{1}{2}{C}_{s}{({\Delta }{\rm{V}})}^{2}\times 3.6$$

4 $${P}_{s}={\rm{3600}}\times \frac{{E}_{s}}{t}$$

where *ΔQ* (C); is the charge stored (expressed in coulombs); *E*_*s*_ is the energy density (Wh·kg^−1^); *P*_*s*_ is the power density (W·kg^−1^); *Cs* is the specific capacitance (F g^−1^).

As shown in Fig. 7f, the energy and power densities of MCMNA-2//AC ASC compared with other cobalt-based spinel-type materials in the Ragone plot. The MCMNA-2//AC ASC indexes the high energy density at 576.0 W·kg^−1^ is 93.1 Wh·kg^−1^. The energy densities keeping up at 67.2 Wh·kg^−1^ at 5760.0 W·kg^−1^. The excellent electrochemical property of MCMNA-2//AC ASC via GCD tests and the data indexed in Fig. 7f. The energy densities (shown in the Fig. 7f) are 93.1 (at 576.0 W·kg^−1^), 89.3 (at 1152.0 W·kg^−1^), 80.6 (at 1728.0 W·kg^−1^), 75.0 (at 2880.0 W·kg^−1^), 71.3 (at 4320.0 W·kg^−1^) and 67.2 Wh·kg^−1^ (5760.0 W·kg^−1^) at 0.2, 0.4, 0.6, 1, 1.5 and 2 A g^−1^, respectively (shown in Table S2).

This MCMNA-2//AC ASC device has higher energy density than other MgCo_2_O_4_ materials which was reported, such as the energy density of the MgCo_2_O_4_//AC of 13 Wh·kg^‒1^ (at 449 W·kg^‒1^)^50^, the MgCo_2_O_4_ cuboidal microcrystals of 24 Wh·kg^‒1^(at 252 W·kg^‒1^)^34^, MgCo_2_O_4_@PPy/NF//AC of 33 Wh·kg^‒1^ (at 320 W·kg^‒1^,)^51^, SiCF/MgCo_2_O_4_//SiCF of 41 Wh·kg^‒1^(at 465 W·kg^‒1^)^52^, mAC//MnCo_2_O_4_ is 3 Wh·kg^−1^ (at 6805. W·kg^‒1^)^12^ (details are shown in Table S3).

Compared with normal MgCo_2_O_4_ supercapacitors, a higher energy and power density are shown in the MCMNA-2//AC ASC device, which indicates that the application of the MCMNA-2 asymmetric supercapacitor has alluring prospects.

## Conclusions

The hierarchical core/shell MgCo_2_O_4_@MnO_2_ nanowall arrays on Ni-foam have been prepared by a facile chemical method (two-hydrothermal-steps). The nanostructure of the core/shell MgCo_2_O_4_@MnO_2_ nanowall arrays is regular and dense which provides a high specific area (0.69 cm^3^·g^−1^; 140.04 m^2^·g^−1^). These materials which composite MnO_2_ have better electrochemical properties from the result of electrochemical measurements. The MgCo_2_O_4_@MnO_2_ nanowall shows an excellent electrochemical performance (852.5 F g^−1^ at 1 A g^−1^). The asymmetric supercapacitors are mainly assembled by the MgCo_2_O_4_@MnO_2_ nanowall and the activated carbon. The energy densities of MCMNA-2//AC device can keep up 67.2 Wh·kg^−1^ at 5760.0 W·kg^−1^. The MgCo_2_O_4_@MnO_2_ nanowall arrays on Ni-foam show excellent supercapacitive performance, which have great potential for more research and application in the asymmetric supercapacitors.

### Experimental section

The process of fabrication of the Mg-Co precursor/Ni-foam is shown in Supplementary Information (Page S1).

The abbreviations of the MgCo_2_O_4_ nanowall arrays and the MgCo_2_O_4_@MnO_2_ core/shell nanowall arrays are MCNA and MCMNA, respectively. These abbreviations will use in this paper.

MCNA: The dry Mg-Co precursor/Ni-foam was calcined at 350 °C for 2 h with a ramping rate of 5 °C/min.

MCMNA-1, MCMNA-2 and MCMNA-3: In the second hydrothermal process, 2.5 mmol of potassium permanganate (KMnO_4_) were dissolved into 50 mL deionized water. The Mg-Co precursor/Ni-foam and the KMnO_4_ solution transferred into a 100 mL Teflon lined autoclave was sealed and maintained at 120 °C for 2 h (MCMNA-1), 4 h (MCMNA-2) and 6 h (MCMNA-3), respectively.

The characterization and electrochemical measurements information and the fabrication and measurement of MCMNA//AC asymmetric supercapacitor were shown in supplementary information (Page. S1–S2).

## Supplementary information

Supplementary Info
