# Supplementary material for: Fabrication of hierarchical core/shell MgCo2O4@MnO2 nanowall arrays on Ni-foam as high–rate electrodes for asymmetric supercapacitors
Source: Sci Rep. 2019 Aug 29;9:12557. doi: 10.1038/s41598-019-48931-6 (PMC6715631; doi:10.1038/s41598-019-48931-6)
Supplement: Supplementary file 1 — Supplementary Info [file 41598_2019_48931_MOESM1_ESM.docx]

*Supplementary Information*

Fabrication of hierarchical core/shell MgCo_2_O_4_@MnO_2_ nanowall arrays on Ni-foam as high–rate electrodes for asymmetric supercapacitors

Jiasheng Xu,*^1,2^** Lin Wang*^2^*

*^1^College of Chemistry, Chemical Engineering and Environmental Engineering, Liaoning Shihua University, Fushun, 113001, P.R. China.*

*^2^Liaoning Province Key Laboratory for Synthesis and Application of Functional Compounds, College of Chemistry and Chemical Engineering, Bohai University, Jinzhou 121013, P.R. China.*

*Correspondence and requests for materials should be addressed to J. X. (email:* [*jiashengxu@bhu.edu.cn*](mailto:liuxy@jlu.edu.cn)*)*

Contents

[Characterization and electrochemical measurements information. S1](#_Toc15283652)

[Fabrication of the Mg-Co precursor/Ni-foam S2](#_Toc15283653)

[Fabrication and measurement of MCMNA//AC asymmetric supercapacitor S2](#_Toc15283654)

[Figure S1 S4](#_Toc15283655)

[Figure S2 S5](#_Toc15283656)

[Figure S3 S6](#_Toc15283657)

[Figure S4 S7](#_Toc15283658)

[Figure S5 S8](#_Toc15283659)

[Figure S6 S9](#_Toc15283660)

[Table S1 S10](#_Toc15283661)

[Table S2 S11](#_Toc15283662)

[Table S3 S12](#_Toc15283663)

[References S13](#_Toc15283664)

# Characterization and electrochemical measurements information.

X-ray diffraction (XRD) patterns were recorded on a Rigaku RAD-3C diffractometer (35 kV, 20 mA, Japan) with Cu Ka radiation (λ=1.548 Å) at a scan rate of 5°/min, in the 2θ angles ranging from 10° to 70°. Thermogravimetric analysis (TGA) was conducted in nitrogen atmosphere at a heating rate of 10 °C/min using a thermal analysis instrument (SDT-Q600, TA instruments). The morphology and size of products were measured using a field-emission scanning electron microscope (FE-SEM, JEOL, S-4800FE). Field emission transmission electron microscope (FE-TEM) images and high resolution electron microscope (HRTEM) images were taken on a transmission electron microscope (JEOL LED JSM-6700F microscope, Japan) with an operating voltage of 200 kV. The nitrogen sorption measurements were performed on a Micromeritics ASAP 2420 surface area analyzer by using Brunauer-Emmett-Teller (BET) method at 77 K. Fourier transform infrared. Spectra were recorded on a FTIR spectrometer (KBr disk method; NEXUS) at wave numbers in the range of 400 – 4000 cm−1.

Electrochemical performance of the as-prepared electrodes was determined using a CHI 660D electrochemical workstation in a three-electrode system. Working electrodes were the as-prepared products. The reference electrode and the counter electrode were a saturated calomel electrode (SCE) and platinum (Pt), respectively. Cyclic voltammetry (CV), chronopotentiometry (CP), electrochemical impedance spectroscopy (Nyquist plot), and the cyclic life were all measured in a KOH electrolyte (2 M). The specific capacitance (F g−1) and current density (A g−1) were calculated based on the mass of active materials.

# Fabrication of the Mg-Co precursor/Ni-foam

All the reagents used in the experiments were of analytical grade and used without further purification. In a typical synthesis, 1.0 mmol of cobalt (II) chloride hexahydrate (CoCl_2_·6H_2_O), 0.5 mmol of Magnesium nitrate hexahydrate (Mg(NO_3_)2·6H_2_O), 2.5 mmol of Ammonium fluoride (NH_4_F) and 5 mmol of urea were dissolved into 50 mL deionized water and 50 mL alcohol at room temperature with magnetic stirring to form a clear solution and transferred into a 100 mL Teflon lined stainless steel autoclave. The Teflon lined autoclave was sealed and maintained at 120°C for 4 h and then naturally cooled down to room temperature. The precipitates were collected by filtration, washed several times with distilled water and absolute ethanol, successively, and then dried at 60 °C for 8 h. The first step of the hydrothermal reaction is completed and the Mg-Co precursor/Ni-foam is obtained

# Fabrication and measurement of MCMNA//AC asymmetric supercapacitor

The Activated Carbon (AC) negative electrode was made of 80 wt% reduced graphene oxide, 10 wt% acetylene black and 10 wt% PVDF. The slurry was well mixed and smear on clean Ni foam, and then dried at 60°C for 8 h. The electrolyte of the supercapacitors was prepared using poly vinyl alcohol (PVA) and KOH. 1 g PVA were dissolved in 20 mL KOH solution (2 M) to get the 5 wt% PVA/KOH gel. The AC on Ni-foam (as the negative), MCMNA-2 on Ni-foam (which is positive), the PVA/KOH gel (as the electrolyte) assembled the asymmetric supercapacitor (ASC) device. The separator is one pieces of cellulose paper in the gap between MCMNA-2 and AC on Ni foam. The electrodes of the ASC device and the cellulose were dipped in KOH solution (2 M) 10 hours. Then the electrodes was mixed with the PVA/KOH gel electrolyte for 15 min to full contact. Assembled the ASC device into a sandwich structure (the illustration of the MCMNA-2//AC device was shown in Fig. 7a). The electrochemical tests were measured by the electrochemical workstation (CHI 660D)

In order to the relationship Q^+^ = Q^−^, the mass of active materials balancing is show as the following formula (1):

$\frac{\text{m}_{\text{+}}}{\text{m}_{\text{}}}\text{= }\frac{{\text{C}_{\text{s}}}_{\text{}}\text{ × }\text{∆V}_{\text{}}}{{\text{C}_{\text{s}}}_{\text{+}}\text{ × }\text{∆V}_{\text{+}}}$ (1)

The mass of the positive electrode surface active material was obtained by measuring the mass change before and after the reaction of the nickel piece and taking the average weight *m*.

According to the calculation, we covered the mixture need on Ni foam. After the vacuum drying the Ni-foam sheet, tableting the sheet under the 5kPa.


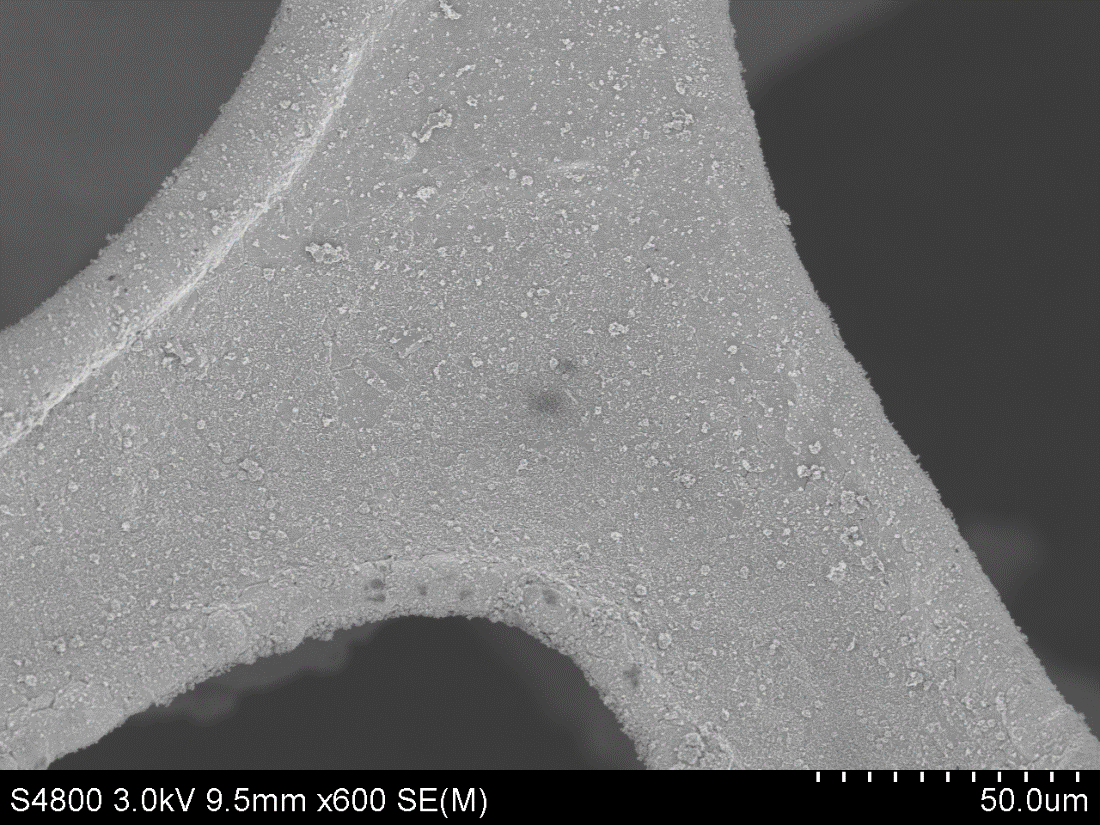


Figure S1 Low magnification SEM image of the MCNA on Nickel foam.


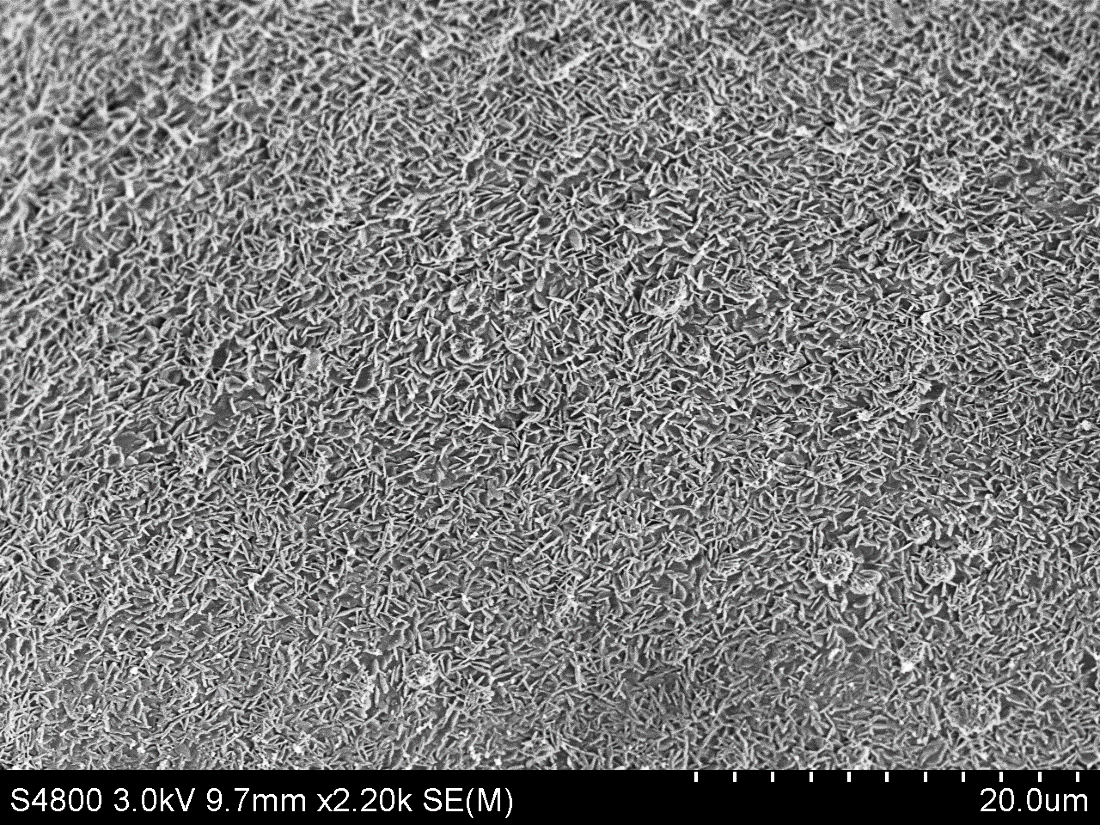


Figure S2 Low magnification SEM image of the MCMNA-2 on Nickel foam


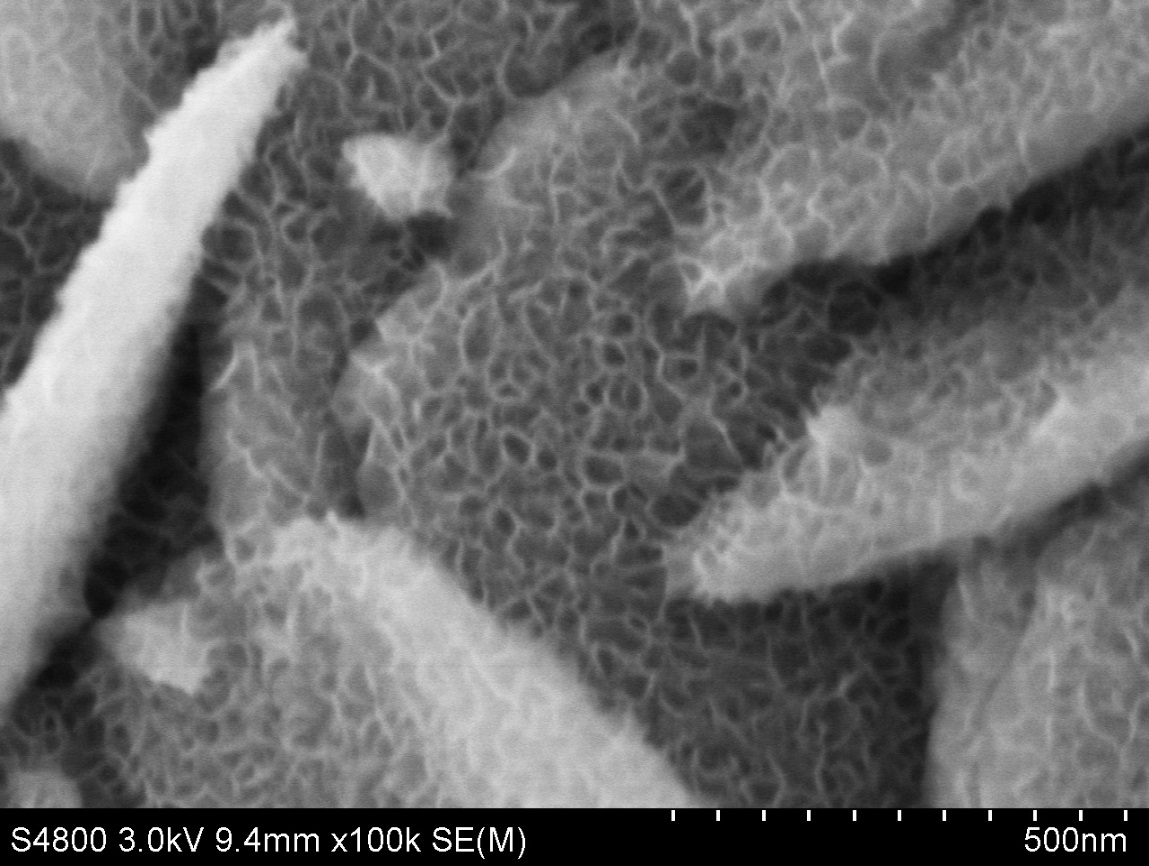


Figure S3 High magnification SEM image of the MCMNA-2 on Nickel foam.

Figure S4 XPS spectrum of the MgCo_2_O_4_@MnO_2_ core-shell nanowall arrays.

Figure S5 Nitrogen adsorption/desorption isotherms of as-prepared hierarchical MgCo_2_O_4_@MnO_2_ core-shell nanowall arrays (MCMNA-2). The inset is the corresponding pore size distribution (measured environment: nitrogen (99.999%) and helium at liquid nitrogen baths of 77.35 K).

Figure S6 Cyclic voltammetry curves of Ni-foam and MCMNA-2 at 40 m Vs^-1^.

Table S1 Capacitive performance of the reported MgCo_2_O_4_ materials as electrode for supercapacitors at three-electrode system.

| Electrode material | Electrolyte | Volage window | Specific capacitors | Ref. |
| --- | --- | --- | --- | --- |
| MgCo_2_O_4_ | 3 M LiOH | 0-0.5 V (vs. Ag/AgCl) | 320.0 F/g (0.5 A/g) | [[1](#_ENREF_1)] |
| MgCo_2_O_4_ NCAs | 6 M KOH | 0-0.5 V (vs. Ag/AgCl) | 750.0 F/g (1 A/g) | [[2](#_ENREF_2)] |
| MgCo_2_O_4_ cuboidal microcrystal | 3 M LiOH | 0-0.5 V (vs. Ag/AgCl) | 690.0 C/g (1 A/g) | [[3](#_ENREF_3)] |
| MgCo_2_O_4_ nanowire | 6 M KOH | 0-0.4 V (vs. Ag/AgCl) | 500.0 F/g (50 A/g) | [[4](#_ENREF_4)] |
| double-urchin-like MgCo_2_O_4_ | 2 M KOH | 0-0.5 V (vs. Hg/HgO) | 508.0 F/g (2 A/g) | [[5](#_ENREF_5)] |
| SiCF/MgCo_2_O_4_ | 1 M KOH | 0-0.6 V (vs. Ag/AgCl) | 203.7 F/g (5 mV/s) | [[6](#_ENREF_6)] |
| SiCF/MgCo_2_O_4_ | 1 M KOH | 0-0.6 V (vs. Ag/AgCl) | 516.7 F/g (5 mV/s) | [[7](#_ENREF_7)] |
| MgCo_2_O_4_ | 3M LiOH | 0-0.5 V (vs. Ag/AgCl) | 355.0 F/g (2.5 mA/cm^2^) | [[8](#_ENREF_8)] |
| MgCo_2_O_4_/rGO | 3M LiOH | 0-0.5 V (vs. Ag/AgCl) | 600.0 F/g (2.5 mA/cm^2^) | [[8](#_ENREF_8)] |
| MCNA | 2 M KOH | 0-0.5 V (vs. Hg/HgO) | 187.4 F/g (1 A/g) | This work |
| MCMNA-2 | 2 M KOH | 0-0.5 V (vs. Hg/HgO) | 852.5 F/g (1 A/g) | This work |

Table S2 Special capacity performance of the reported MgCo_2_O_4_ materials as electrode for supercapacitors at two-electrode system.

| I/m（A/g) | 0.2 | 0.4 | 0.6 | 1 | 1.5 | 2 |
| --- | --- | --- | --- | --- | --- | --- |
| Cs (F/g) | 72.8 | 69.8 | 63.0 | 58.6 | 55.7 | **52.5** |
| Specific capacity (C/g) | 931.2 | 446.4 | 268.6 | 150.1 | 95.0 | **67.2** |
| Es (Wh/kg) | 93.1 | 89.3 | 80.6 | 75.0 | 71.3 | 67.2 |
| P_s_ (W/kg) | 576.0 | 1152.0 | 1728.0 | 2880.0 | 4320.0 | 5760.0 |

Table S3 Comparison of the energy density vs. power densities of the assembled the MgCo_2_O_4_@MnO_2_//AC asymmetric supercapacitor device in this work and other reported.

| Electrode material | Energy density (Wh kg^‒1^) | | Power density (W kg^‒1^) | | Ref. |
| --- | --- | --- | --- | --- | --- |
| SiCF/MgCo_2_O_4_//SiCF | 41.3 | 464.7 | | | [[6](#_ENREF_6)] |
| MgCo_2_O_4_//AC | 13.0 | 448.7 | | | [[9](#_ENREF_9)] |
| MgCo_2_O_4_ cuboidal microcrystals | 24.0 | 252.0 | | | [[3](#_ENREF_3)] |
| MgCo_2_O_4_@PPy/NF//AC | 33.4 | 320.0 | | | [[10](#_ENREF_10)] |
| mAC//MnCo_2_O_4_ | 2.6 | 6805.0 | | | [[11](#_ENREF_11)] |
| MCMNA-2//AC | 89.3 | 1152 | | This work | |

# References

1 Krishnan, S.G. *et al.* Characterization of MgCo_2_O_4_ as an electrode for high performance supercapacitors. *Electrochim. Acta* **161**, 312-321 (2015).

2 Cui, L. *et al.* High-performance MgCo_2_O_4_ nanocone arrays grown on three-dimensional nickel foams: Preparation and application as binder-free electrode for pseudo-supercapacitor. *J. Power Sources* **333**, 118-124 (2016).

3 Krishnan, S.G. *et al.* Effect of processing parameters on the charge storage properties of MgCo_2_O_4_ electrodes. *Ceram. Int.* **43**, 12270-12279 (2017).

4 Guan, X. Morphology-tuned synthesis of MgCo_2_O_4_ arrays on graphene coated nickel foam for high-rate supercapacitor electrode. *Int. J. Electrochem. Sci.* 10.20964/2018.03.35, 2272-2285 (2018).

5 Xu, J. *et al.* Fabrication of porous double-urchin-like MgCo_2_O_4_ hierarchical architectures for high-rate supercapacitors. *J. Alloys Compd.* **688**, 933-938 (2016).

6 Kim, M. & Kim, J. Redox active KI solid-state electrolyte for battery-like electrochemical capacitive energy storage based on MgCo_2_O_4_ nanoneedles on porous β-polytype silicon carbide. *Electrochim. Acta* **260**, 921-931 (2018).

7 Kim, M., Yoo, J. & Kim, J. A p-nitroaniline redox-active solid-state electrolyte for battery-like electrochemical capacitive energy storage combined with an asymmetric supercapacitor based on metal oxide functionalized β-polytype porous silicon carbide electrodes. *Dalton Trans* **46**, 6588-6600 (2017).

8 Krishnan, S.G. *et al.* Critical influence of reduced graphene oxide mediated binding of M (M = Mg, Mn) with Co ions, chemical stability and charge storability enhancements of spinal-type hierarchical MCo_2_O_4_ nanostructures. *Electrochim. Acta* **243**, 119-128 (2017).

9 Vijayakumar, S., Nagamuthu, S. & Ryu, K.-S. In-situ preparation of MgCo_2_O_4_ nanosheets on Ni-foam as a binder-free electrode for high performance hybrid supercapacitors. *Dalton Trans.* 10.1039/C8DT00591E, 13-21 (2018).

10 Gao, H. *et al.* An urchin-like MgCo_2_O_4_@PPy core–shell composite grown on Ni-foam for a high-performance all-solid-state asymmetric supercapacitor. *Nanoscale* **10**, 10190-10202 (2018).

11 Shree Kesavan, K., Surya, K. & Michael, M.S. High powered hybrid supercapacitor with microporous activated carbon. *Solid State Ionics* **321**, 15-22 (2018).
